# Vestibular-Evoked Myogenic Potentials in Bilateral Vestibulopathy

**DOI:** 10.3389/fneur.2018.00252

**Published:** 2018-04-17

**Authors:** Sally M. Rosengren, Miriam S. Welgampola, Rachael L. Taylor

**Affiliations:** ^1^Neurology Department, Royal Prince Alfred Hospital, Sydney, NSW, Australia; ^2^Central Clinical School, University of Sydney, Sydney, NSW, Australia; ^3^Institute of Clinical Neurosciences, Royal Prince Alfred Hospital, Sydney, NSW, Australia; ^4^Audiology Department, Whangarei Hospital, Whangarei, New Zealand; ^5^New Zealand Dizziness and Balance Centre, Auckland, New Zealand

**Keywords:** vestibular-evoked myogenic potential, otolith, gentamicin, aminoglycoside, bilateral vestibulopathy, Meniere’s disease, vestibular

## Abstract

Bilateral vestibulopathy (BVP) is a chronic condition in which patients have a reduction or absence of vestibular function in both ears. BVP is characterized by bilateral reduction of horizontal canal responses; however, there is increasing evidence that otolith function can also be affected. Cervical and ocular vestibular-evoked myogenic potentials (cVEMPs/oVEMPs) are relatively new tests of otolith function that can be used to test the saccule and utricle of both ears independently. Studies to date show that cVEMPs and oVEMPs are often small or absent in BVP but are in the normal range in a significant proportion of patients. The variability in otolith function is partly due to the heterogeneous nature of BVP but is also due to false negative and positive responses that occur because of the large range of normal VEMP amplitudes. Due to their variability, VEMPs are not part of the diagnosis of BVP; however, they are helpful complementary tests that can provide information about the extent of disease within the labyrinth. This article is a review of the use of VEMPs in BVP, summarizing the available data on VEMP abnormalities in patients and discussing the limitations of VEMPs in diagnosing bilateral loss of otolith function.

Bilateral vestibulopathy (BVP) is a rare and chronic condition resulting from a loss or reduction of vestibular function in both ears (1, 2). As vestibular function is critical for maintaining balance and holding gaze steady during movement, absence of vestibular function causes disabling unsteadiness and oscillopsia (3). The unsteadiness becomes worse in the dark and when walking on uneven ground due to reduction in vision and proprioception, which are key contributors to balance in these situations. In contrast, patients are typically free of symptoms when they sit with their head still. BVP can also have effects on cognition, including visuospatial ability. Patients with BVP sometimes have no other neurological deficits or hearing loss, apart from presbycusis. BVP has many causes, such as exposure to ototoxic drugs (e.g., aminoglycosides), infections (e.g., meningitis), autoimmune disease, genetic disorders (e.g., Usher syndrome or DFNA9), and Meniere’s disease, while a significant number of cases are idiopathic (1, 4, 5).

Demonstration of vestibular loss has historically been sought by a caloric or rotational chair test, both of which assess the horizontal angular vestibulo-ocular reflex (VOR) (6, 7). More recently, video systems for recording the head impulse test (vHIT) have become more widely available, allowing measurement of the VOR from all three canals in both ears (8). These are all tests of semicircular canal function, and all but the vHIT of the posterior canal are tests of the superior vestibular nerve. Indeed BVP is characterized by bilateral reduction of horizontal canal responses. There is, however, increasing evidence that otolith function can also be affected in BVP. For example, in 1997 Lempert et al. (9) showed that some patients with BVP had abnormal otolith-ocular reflex gain, symmetry and/or latency, and concomitant deficits in dynamic visual acuity during lateral translations, suggesting abnormalities of the otolith organs.

In recent years, vestibular-evoked myogenic potentials (VEMPs) have become a widespread test of otolith function (10). Cervical VEMPs (cVEMPs) are short-latency inhibitory reflexes recorded from the sternocleidomastoid (SCM) muscle, while ocular VEMPs (oVEMPs) are excitatory reflexes recorded from the inferior oblique extraocular muscles. VEMPs are considered tests of otolith function because the brief bursts of air-conducted (AC) sound or bone-conducted (BC) skull vibration used to produce them have been shown to preferentially activate irregularly firing otolith afferents in both rats and guinea pigs (11, 12). The cVEMP produced by an AC sound stimulus is a test of the saccule as this organ has the lowest threshold to AC sound stimulation and because the projection to the SCM muscle in humans (an ipsilateral inhibition) matches the projection shown in animal studies (13, 14). Likewise, the oVEMP produced by either stimulus is thought to be predominantly utricular because the contralateral excitatory projection to the inferior oblique muscle in humans matches that seen in animals (14, 15). For both reflexes, studies in patients with vestibular neuritis, who have relatively selective lesions of the superior or (rarely) inferior vestibular nerves, support an origin in the inferior (cVEMP) and superior nerves (oVEMP) (16). VEMPs are particularly useful in BVP as they remain abnormal after central vestibular compensation has occurred and can test the ears independently, unlike tests of subjective visual vertical or horizontal, which reveal only unilateral otolith abnormalities in the acute phase of disease.

## Characteristics of cVEMPs and oVEMPs in BVP

Cervical VEMP abnormalities in BVP were first reported by Matsuzaki and Murofushi (17), who tested three patients who had absent ice water caloric responses bilaterally. They found that cVEMPs were absent in five of the six ears, suggesting that the saccule and inferior vestibular nerve were also affected by the disease. A further two patients reported by the same group had unilateral cVEMP abnormalities using both AC sound and galvanic vestibular stimulation (18). In 2003, Brantberg (19) described a family with presumed early-onset vestibulopathy, in which a father and two sons had attenuated caloric responses and the father additionally had absent AC cVEMPs. Brantberg hypothesized that the vestibulopathy affected the canals before the otoliths but did not extend to the cochlea. However, in a subsequent article, Brantberg and Löfqvist (20) presented a series of five patients with symptoms of unsteadiness and oscillopsia and absent caloric responses who were diagnosed with idiopathic BVP. They found that, although one patient had asymmetric amplitudes, all five patients had well-formed cVEMPs bilaterally, suggesting that saccular function may be largely spared in BVP.

Several early oVEMP studies reported absent BC oVEMPs in small series of patients, suggesting possible utricular involvement in BVP (21–23). However, these patients were recruited because they were known to have absent cVEMPs as well as absent caloric responses (to demonstrate the vestibular dependence of oVEMPs) and may not be representative of BVP patients in general. Chiarovano et al. (24) recorded oVEMPs in response to AC sound stimulation in a wide variety of patients and reported absent oVEMPs in nine patients with BVP due to aminoglycoside ototoxicity.

Two relatively large studies have now shown that cVEMPs and oVEMPs can indeed be small or absent in BVP, but in fact fall in the normal range for a significant proportion of patients. Zingler et al. (25) recorded AC cVEMPs in 84 patients with complete or partial BVP and found that cVEMPs were significantly smaller in the patients than controls (by approximately 35%). However, there were only four patients with an absent cVEMP unilaterally and no patients with absent responses bilaterally. In contrast, all 40 patients had absent caloric responses bilaterally. Agrawal et al. (26) recorded both AC cVEMPs and BC oVEMPs in 34 patients with BVP. VEMPs were considered abnormal if they were absent or the amplitude was below the fifth percentile of the normal control group. Using this criterion, 61% of patients had abnormal cVEMPs and 64% had abnormal oVEMPs. However, as the control group was much younger than the patient group, and VEMPs tend to decline with age (the AC cVEMP more than the BC oVEMP), this might be an overestimate of the rate of abnormalities. The number of patients with absent responses was not reported. Caloric slow phase velocity SPV was not correlated with cVEMP amplitude in either of the above studies, however, it was correlated with oVEMP amplitude (*r* = 0.51) (26), consistent with the caloric and oVEMP both being tests of superior vestibular nerve function.

## Concordance of Otolith and Canal Function in BVP of Different Etiologies

Zingler et al. (25) found no differences among patients with different etiology or clinical course (progressive or sequential) of BVP. In contrast, Agrawal et al. (26) compared patients with BVP due to aminoglycoside toxicity, MD, and mixed origins. They found that patients with aminoglycoside toxicity tended to have the smallest responses on both the caloric and VEMP tests, though the difference between etiologies only reached significance for the oVEMP when comparing aminoglycoside toxicity and MD. It thus appears that systemic aminoglycoside toxicity has relatively severe effects across all vestibular organs (24, 26). This is not surprising, as studies of topical application of gentamicin for the treatment of intractable Meniere’s disease have shown significant deterioration of cVEMPs (27–29). BVP caused by bilateral Meniere’s disease is also likely to be associated with significant bilateral saccular abnormalities (26), as unilateral MD is associated with specific AC cVEMP abnormalities (30). However, caution is required when considering MD together with other causes of BVP. MD has a characteristic pattern of vestibular and auditory deficits, with significant levels of cVEMP abnormality, and inclusion of patients with MD may inflate the rate of cVEMP abnormalities compared to other causes of BVP.

A surprising frequency of preserved VEMPs has also been reported in patients with BVP combined with cerebellar atrophy. Marti et al. (31, 32) described five patients with cerebellar atrophy and bilateral vestibulopathy (CABV), now renamed cerebellar atrophy, neuropathy, and vestibular areflexia syndrome (CANVAS), who had preserved AC cVEMPs and ocular counterroll responses. In a later study of 31 patients with CANVAS, only 7 had absent AC cVEMPs, while 17 had impaired caloric responses and all had a bilaterally positive bedside HIT (33). Finally, a recent case report described a patient with CANVAS with preserved AC cVEMPs and oVEMPs, but absent caloric and rotation responses and absent vHIT responses in all six canal planes (34).

The dissociation of canal and otolith function is even more obvious in patients with bilateral vestibular loss due to large vestibular aqueduct syndrome. A recent article showed that many patients had bilateral canal paresis on caloric testing, but augmented AC cVEMPs and oVEMPs, with enlarged amplitudes and lowered thresholds compared to controls (35). However, the patients in this study were 7–27 years of age and these findings of canal hypofunction-otolith hyperfunction may not be applicable to older patients with LVAS.

## Possible Causes of the Lower Prevalence of Otolith Dysfunction in BVP

**Figure 1:**
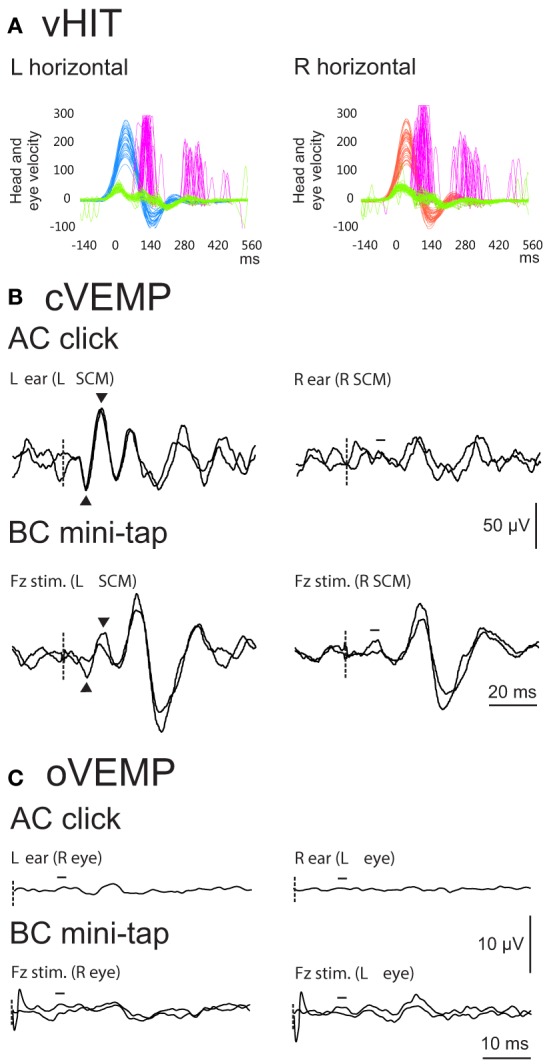
Example vHIT **(A)**, cervical vestibular evoked myogenic potential (cVEMP) **(B)** and ocular VEMP (oVEMP) **(C)** results from a 54-year-old male patient with idiopathic bilateral vestibulopathy (BVP). For cVEMPs and oVEMPs, stimulus onset is indicated by the dashed line. vHIT results show reduced horizontal vestibulo-ocular reflex (VOR) gain [shown by the gap between the green (eye) and blue (head) traces on the left, and green and red traces on the right] and the presence of catch-up saccades (purple traces) on both sides. VOR gain on the left was 0.39 and on the right was 0.17, indicating that the patient met the test criteria for a diagnosis of BVP (gain less than 0.6) (6). cVEMPs evoked by air-conducted (AC) sound were clearly present on the left but absent on the right. cVEMPs evoked by bone-conducted (BC) mini-taps were present on one trial on the left only but were not readily reproducible. oVEMPs evoked by both AC and BC stimulation were absent bilaterally. The results in this patient highlight the mixed results often seen with VEMP testing in BVP.

One reason for the canal-otolith dissociation in BVP relates to the diagnostic criteria. By definition, all of the patients have profoundly abnormal or absent horizontal semicircular canal function bilaterally as measured by the caloric and/or HIT. It is therefore to be expected that horizontal canal dysfunction is universal in BVP, while all other end organs may have lesser degrees of dysfunction (see Figure 1 for example). The Barany society has recently published a consensus document on the diagnostic criteria for BVP (6). To receive a diagnosis, patients must have a chronic clinical syndrome consisting of unsteadiness when standing or walking, combined with oscillopsia during head or body movements and/or worsening of unsteadiness in the dark or on uneven ground. They must also have bilaterally reduced or absent angular VOR function documented by vHIT, caloric, or rotational testing. VEMPs, and other tests of otolith function, remain peripheral to the diagnosis of BVP as they are not reliably abnormal (6).

A potential problem with the exclusion of otolith function from the diagnosis of BVP is that patients who may present with disease affecting predominantly the otolith organs would be missed. In fact, several studies have proposed a rare type of BVP, which affects the inferior vestibular nerve and causes abnormal cVEMPs, but spares the superior vestibular nerve (36–38). However, the patients in these studies were identified retrospectively from large databases by their abnormal AC cVEMP results and not by their presenting symptoms. It is currently not known whether isolated bilateral otolith dysfunction causes significant disability. Given that cVEMPs are known to be absent occasionally in normal subjects (more so with increasing age), this type of study design makes it difficult to distinguish the effects of disease from a false positive (abnormal) test result. It is possible that these cases simply represent the false positive rate expected for the cVEMP test. Further studies are therefore needed to confirm the existence of an isolated otolithic BVP, ideally using additional tests to confirm the otolith loss [such as eccentric rotation or measurement of the ocular tilt reflex (31)].

The variability of otolith function in BVP is very likely to depend on the cause of BVP, as mentioned above. Bilateral loss of vestibular function is the final outcome of a range of diseases with variable course and duration, including exposure to ototoxic drugs, neurodegenerative disease (CANVAS), and congenital malformations (LVAS). As it is a rare condition and VEMPs are relatively new tests, there are few studies comparing sufficient numbers of patients with different etiologies. Alternately, factors relating to the VEMP test itself may also affect the rate of otolith abnormalities found in BVP.

Due to the large range of normal VEMP amplitudes false negative responses may occur, i.e., responses fall in the normal range, but may be smaller than they would be without the effects of BVP. Unlike the vHIT, in which the obtained VOR gain is compared to an ideal of 1.0 (39), there is no objective target amplitude for VEMPs. While unilaterally reduced amplitudes are easy to detect by comparison with the opposite side, bilaterally reduced amplitudes can usually be detected only in group comparisons and not in individual patients. This can be seen in studies of BPV, in which average patient amplitudes are smaller than control values (25, 26), suggesting a degree of otolith dysfunction. VEMP amplitude is determined not only by the effect of the stimulus on the otolith organs but also by measurement of a synchronous change in motor activity in the target muscle. It is not known how much residual otolith activity is required to produce a synchronous motor discharge or how much otolith function can be lost before a VEMP falls below the 5% normal limit.

False positive (abnormal) responses can also occur for several reasons. There is a well-documented effect of age on reflex amplitudes, which is greater for the AC cVEMP than BC oVEMP (40–44). For the AC cVEMP, the range of normal amplitudes extends down to an absent response in older age groups (41, 45, 46). There is only a small effective window for AC stimulus intensity: between the vestibular threshold for AC sound and the safe upper limit of cochlear sound exposure. Factors such as conductive hearing loss, aging, and weak stimulus intensity can all shift an ear out of this effective range (and may affect the ears reasonably symmetrically, producing bilateral test abnormalities). In addition, for the cVEMP, muscle contraction strength and its measurement are also important considerations (47–50). Very weak contractions may erroneously lead to absent or abnormal responses (50). For the oVEMP, angle of vertical gaze and stimulus type are important factors (51). BC stimulation produces robust oVEMPs that are only mildly affected by age, while AC stimulation produces very small responses that are significantly affected by age and often absent, leading to high rates of abnormality in normal subjects (41). For both reflexes, it is important to ensure results are reliable and valid before a decision is made about normality/abnormality. Apart from using correct stimulation and recording techniques, a major factor is a good signal-to-noise ratio, which can be optimized by comparing fewer, longer recordings for each ear, rather than multiple short recordings, and obtaining a relatively flat prestimulus baseline. It is also important for laboratories to have their own normal data, particularly in the upper age ranges. However, even with good normal data, it can be theoretically problematic to define a lower limit of normal, as bilaterally small or absent responses are a normal finding in older patients. Agrawal et al. (26) defined abnormal amplitudes as those below the fifth percentile of normal control data, which is reasonable as it assumes the same level of error as commonly applied in statistical analysis. Similar problems with false positives and negatives can also be ascribed to the caloric test and there are different conventions across laboratories regarding the lower limits of normal SPV (6).

## Conclusion

Vestibular-evoked myogenic potential studies have shown a range of otolith function in patients with BVP. This variability can be partly attributed to the heterogeneous nature of BVP but is also due to the nature of the VEMP tests and the large range of responses present in normal subjects. It is appropriate that VEMPs remain a complementary test in BVP: while not helpful to the diagnosis of BVP, VEMP-vHIT-caloric dissociations may prove useful in determining its etiology and provide information about the extent of disease within the labyrinth. They may also be helpful in monitoring disease progression and guiding rehabilitation. As vHIT and VEMPs become more widespread, we hope to see more large studies of BVP patients with different etiologies to better understand the effects of BVP on canal and otolith function.

## Author Contributions

SR drafted and edited the manuscript. MW and RT edited the manuscript.

## Conflict of Interest Statement

The submitted work was not carried out in the presence of any personal, professional or financial relationships that could potentially be construed as a conflict of interest.
